# Psychiatric Discharge Process

**DOI:** 10.5402/2012/638943

**Published:** 2012-09-04

**Authors:** Hamzah M. Alghzawi

**Affiliations:** Faculty of Nursing, The Al-Albayt University, Mafraq 130040, Jordan

## Abstract

*Background.* Integration of research evidence into clinical nursing practice is essential for the delivery of high-quality nursing care. Discharge planning is an essential process in psychiatric nursing field, in order to prevent recurrent readmission to psychiatric units. *Objective.* The purpose of this paper is to perform literature overview on psychiatric discharge planning, in order to develop evidence-based practice guideline of psychiatric discharge plan. *Methods.* A search of electronic databases was conducted. The search process aimed to locate different levels of evidence. Inclusion criteria were studies including outcomes related to prevention of readmission as stability in the community, studies investigating the discharge planning process in acute psychiatric wards, and studies that included factors that impede discharge planning and factors that aid timely discharge. On the other hand, exclusion criteria were studies in which discharge planning was discussed as part of a multi faceted intervention and was not the main focus of the review. *Result.* Studies met inclusion criteria were mainly literature reviews, consensus statements, and descriptive studies. All of these studies are considered at the lower levels of evidence. *Conclusion.* This review demonstrated that discharge planning based on general principles (evidence based principles) should be applied during psychiatric discharge planning to make this discharge more effective. Depending on this review, it could be concluded that effective discharge planning includes main three stages; initial discharge meeting, regular discharge meeting(s), and leaving from hospital and discharge day. Each stage of them has requirements should be accomplished be go to the next stage.

## 1. Introduction

Discharge planning is a vital process in nursing field. Discharge planning could be defined as a dynamic, comprehensive, and collaborative process that should be started at the time of admission and its purpose is to identify the client's plans and the support which the client and caregiver would require after existing from psychiatric unit [1].

In the health care field “discharge planning” is one of the most important issues in our time, it is at once a methodology, a discipline, a function, a movement, and a solution [2]. 

## 2. Significance and Purpose of the Paper

By increased pressures for rapid discharge of psychiatric patients as a result of various government cost containment strategies, it is essential that a comprehensive discharge planning process be established in psychiatric facilities [2].

Discharge planning is widely considered as a necessary component in the community care of the chronic mentally ill. The new trends of deinstitutionalization were started with no comprehensive planning for adequate or appropriate community resources [3], this means that discharge planning must be creative.

The purpose of this paper is to perform literature overview on psychiatric discharge planning, in order to develop evidence-based practice guideline of psychiatric discharge plan. So, what is the meaning of psychiatric discharge planning, what is the importance of discharge planning, who is involved in psychiatric discharge planning, what are the considerations discharge planning based on, and does effective psychiatric discharge planning prevent readmission to inpatient psychiatric units? Answers for these questions and others will be searched in the literature.

## 3. Methodology

### 3.1. Search Strategy

All levels of evidence as defined by the NHMRC (2000) were searched, the search strategy aimed to locate different study designs such as systematic reviews and meta-analyses, randomized controlled trials, controlled trials, cohort or case-control analytic studies, expert opinion including literature/narrative reviews, consensus statements, descriptive studies, and individual case studies.

### 3.2. Key Search Words

Clients, psychiatric unit, mental health unit, acute, discharge, discharge plan, discharge process, and prevention of readmission.

### 3.3. Inclusion/Exclusion Criteria

Inclusion criteria were studies including outcomes related to prevention of readmission as stability in the community, studies investigating the discharge planning process in acute psychiatric wards (assessment on admission, inpatient assessment, preparation of individualized discharge plan, provision of interventions, monitoring), and studies that included factors that impede discharge planning and factors that aid timely discharge. On the other hand, exclusion criteria were studies in which discharge planning was discussed as part of a multifaceted intervention and was not the main focus of the paper.

## 4. Literature Review

Discharge planning is an important step in order to maintain gains achieved during the course of treatment that the clients have in the inpatient psychiatric unit. On the other hand, lack of discharge planning can cause the disturbance in the care of the client which is considered as one of the most significant obstacles to establishing a stable recovery [4].

The client after discharge may still be in need of self-help groups, relapse prevention groups, continued individual counseling, and mental health services especially important for clients who will continue to require medication, as well as intensive case management monitoring and support [5]. So, a carefully developed discharge plan, produced in collaboration with the client, will identify and match client needs with community resources, providing the support needed to sustain the progress achieved during treatment.

Numerous concepts that could be used to improve the effectiveness of discharge planning became apparent. First, because there are usually multiple health providers for a single patient, continuity of care can be achieved only by interventions to establish personal and specific linkages between the discharging facility and the aftercare provider [6]. Second, to ensure the relevancy of the referral to outpatient care setting, inpatient treatment should include the coordination of community services that are matched to the patient's level of functioning [6]. Third, “nonpsychiatric obstacles,” such as housing, employment, and need for supplemental security income, that serve as difficult barriers to effective planning must be dealt with [7]. And, finally, the discharge plan must be integrated into the treatment process in such a way that the patient is offered the chance to become an active participant in the plan and thus is more likely to accept it [6].

 Health providers in the community support system noted that many of the patients who were resistant to aftercare services were those for whom discharge planning came late during their hospitalization, or for whom plans were not integrated into the treatment process; however, plans were made early [6].

Moore [8] conducted a study about discharge from an acute psychiatric ward. This descriptive study aimed to find out whether “discharge is planned to support improvement of symptoms and prevent future readmissions.” However, little information is given about how the study was carried out. It was found an improvement in patient symptom during admission.

Cohen et al. [9] conducted a study to examine the factors that influence the inpatient team's ability to secure a “good enough” fit between the patient's needs and an optimal discharge plan. 494 consecutive admissions had the Mount Sinai Discharge Planning Inventory completed weekly during admission. Discharge planning was able to have greater impact in the areas of increase daily activities and establishing relevant treatment options. Assisting patients to find a more suitable living arrangement was an area that discharge planners had greater difficulty with.

The authors advocate the Discharge Planning Inventory as a tool to track progress and evaluate discharge planning. It is stated that the optimal first choice discharge plan was “identified by a consensus among professional clinician's based upon patient's needs” [9, page 520]. This implies that there was little input from patients in identifying their own needs for discharge. Similarly the Discharge Planning Inventory was completed by a social worker with apparently little input from patients.

Another study by Caton and associates [10] showed that the quality of discharge planning was predictive of rehospitalization within three months when the patient's prognosis was taken into account. Also, Caton and Gralnick [11] reviewed the literature about the factors which affect length of psychiatric hospitalization and concluded that rather than diagnosis itself, other environmental and delivery systems factors together may have predictive ability [12]. Access to environmental supports is felt to significantly influence recovery from psychiatric illness [13]. Really, such factors as housing and placement considerations, level of social competence or functioning, severity of psychiatric condition, and adequacy of social supports have been reported to contribute to the length of inpatient stay [14]. Furthermore, family involvement, continuity of care, psychosocial rehabilitation, psychoeducation, selection of appropriate medications, and patient cooperation have been identified as factors contributing to after discharge recovery [15].

 Rock [16] described the Expert viewpoint about the essential elements in providing quality discharge planning services. He recommended that discharge planning must be a collaborative effort including all clinical departments. Also, Discharge planning process need to be supported by effective posthospital support programs.

Altman [6] examined the use of collaborative discharge planning (CDP) meetings for patients with chronic mental illness patients. A higher percentage of patients who were involved in collaborative discharge planning became involved in aftercare services compared to those who were not involved in CDP. A collaborative process between hospital staff, the patient, the family, and community agencies lead to advocates discharge planning. But in this study little demographic information is given about the 2 compared groups or how patients were allocated to the groups. Also sample is relatively small (29 patients).

Ledbetter and Batey [17] described a resource group model. This service user education model was used in a small group setting to provide information about medications, community resources, and vocational rehabilitation services. The authors propose that this model encourages involvement of clients in discharge planning, facilitation of interpersonal skills, and integration of services. This was an interesting outline of group work in an inpatient setting but was limited in the discussion and analysis of outcomes.

Kelly et al. [18] examined factors in delays in discharge from acute-care psychiatry and threw a survey of 327 patients from 12 psychiatry units. It included the use of the Brief Psychiatric Rating Scale (BPRS) and the Discharge Readiness Inventory (DRI). There was followup at 30 days to determine discharge outcome. Both the BPRS and the DRI were altered for use in this study, which may have affected the reliability and validity of the instruments. In this study, a proportion of patients who were (clinically) ready for discharge were not discharged due to ongoing behavior and medication stabilization and lack of community resources such as housing. This means that improving access to residential placement would reduce length of stay for some patients. Also, Patients whose discharge was delayed were found to have higher levels of conceptual disorganization, hallucinations, disorientation, and more active symptoms. These patients could be targeted for early intervention and early discharge planning. Giving perception of discharge from the perspective of the staff members completing the survey was identified and discussed in this study as clinical implications and limitations.

Buckwalter [19] described methods that should be used in predischarge planning programs to assist patients “take charge of their illness and become partners in the treatment process” (page 15) and reduce likelihood of readmission. The main components that should be considered in discharge planning according to the researcher are working with the patients' family, giving the patient simple and accurate literature to read about their illness, assisting patients to understand what their diagnosis means, assigning homework that requires the patient and family to read instructional material between appointments, assisting patients in looking at their stressors and exploring stress management techniques, assisting patients to recognize the meaning of their symptoms, assisting patients to develop ways of explaining their hospitalization when discharged, encouragement to continue with recreational activity, and education about medication and encouragement of compliance. However these methods appear effective, they reflected the opinion of two clinicians from one discipline and so may be a limited representation of the issues related to discharge planning.

Caton et al. [10] studied the impact of discharge planning on chronic schizophrenic patients. This study conducted on 114 patients with chronic schizophrenia at 4 inpatient psychiatric units. The discharge planning schedule was developed for this study and involved interviewing patients, staff and family. The community care schedule was then administered to patients three months after discharge. A study of the interrater reliability of the discharge planning schedule and the community care schedule was carried out. The adequacy of discharge planning bore no significant relationship to role functioning, daily activities, social isolation, or employment at 3 months after discharge. Patients who had adequate discharge planning for vocational issues were not more likely to attend vocational rehabilitation or participate in the labour force. Patients who received adequate discharge planning for aftercare services were more likely to comply with aftercare treatment and were less likely to be readmitted. Discharge planning for living arrangements was based on what was available and the patient's financial resources rather than on what might have been most desirable for successful community living.

The literature review on the information needed at discharging patients from hospital is wide. Literatures have shown that this information should include the differential diagnosis, management, treatment on discharge, prognosis, what the patient and relatives were told, and future management plans including details of the responsibilities of all involved as well as the date of any follow-up appointment [1]. Moreover, another study concluded that information should include: dates of admission and discharge; how the patient came to be admitted; type of admission; diagnosis and management; type of discharge; disabilities on discharge; what patient and relatives were told; prospects for returning to work (work prognosis); accommodation on discharge; medication, drugs, dosage, frequency, and quantity; risk factors that increase the need for followup; follow-up plans and responsibilities; services, and facilities organized; whether or not a shared care record was given [20].

The discharge summaries provided valuable data which is easy to retrieve and analyze. This summary is potentially a valuable document for the psychiatric team, providing detailed information concerning a patient's hospital admission, previous clinical history, and potential risk factors for the patient and those involved in his or her care [21]. There is evidence that using a structured summary helps to focus on the most appropriate information, facilitates recovery, has educational value, and promotes briefness [22].

## 5. Conclusion and Recommendations

Discharge planning should begin upon client admission into the psychiatric unit and the discharge plan should continue to be updated during the course of the client's treatment stay with the provider. The benefits of conducting discharge planning for clients with mental illness are to link clients to appropriate next step resources based on their needs; to minimize likelihood that client will “relapse” or have to return to care post successful completion of treatment; to prevent vulnerable clients from becoming homeless and/or criminalized; to assist clients with re-entry to community.

Patients with major mental disorders appear to continue to need readmission to psychiatric institution for stabilization despite the accessibility of social resources in their community. Even so, discharge planning still important in assessing the needs of patients for social, rehabilitative, and specialized services to achieve the goal of improving the quality of life of the vast majority of patients who have required hospital admission.

Discharge planning should be a collaborative process between hospital staff, the patient, the family, and the community aftercare agencies so that vital linkages are affected before discharge. The literature demonstrated the usefulness of a collaborative model to inpatient staff who, until the wave of deinstitutionalization overtook them, was more accustomed to managing the acutely ill patient.

Discharge planning is a team approach that should include the client and, when appropriate, family members. Generally, the treatment team should include psychologist, social worker, psychiatrist, case manager, vocational specialist, and housing professionals who should participate in creating the discharge plan. In addition, the team should include the community partners of the client, such as peers, relatives, and friends.

Literature review about discharge planning recommended that discharge planning should be tailored for different needs of different client, be comprehensive which mean address client's need across multiple health system in the plan, create a system that is continuous and coordinated, be practical and realistic, and maximize available resources for the benefit of the client.

The discharge summaries are important part of the discharge process because they provided valuable data about the clients which is easy to recall up in order to facilitating client's followup in the community.

## 6. Application of Discharge Planning

### 6.1. Applicable Guideline

#### 6.1.1. Introduction

Discharge planning is defined as a dynamic, flexible, comprehensive, and collaborative process that should be started at the time of admission and its aim is to identify the client's plans and needs to support them after existing from psychiatric unit.

Literature review demonstrated that discharge planning based on general principles (evidence-based principles) should be applied during psychiatric discharge planning to make this discharge more effective.

Also, depending on literature review, it could be concluded that effective discharge planning includes main three stages initial discharge meeting, regular discharge meeting(s), and leaving from hospital and discharge day. Each stage of them has requirements that should be accomplished to go to the next stage.

The length of time between the first stage and the final stage of discharge planning depends upon the progress of the clients. This means that the second stage of regular meeting may need more than one meeting based on patient progress.

## 7. General Principles for Discharge (Applicable Principles)

- Careful planning is the corner stone of successful care in the community. Discharge planning will form part of the assessment and care planning process with a patient on admission to an inpatient unit.
- Care teams, in collaboration with the patient and their carer's (mostly the family), will formulate a discharge care plan and risk and relapse plan relating to the specific needs of the individual.
- Planning for discharge should begin as soon as possible following admission in order that a comprehensive treatment and discharge plan can be formulated.
- The patient and carer, should be fully involved in all aspects of the discharge plan, where practicable. Arrangements for discharge should be negotiated with everyone likely to be concerned with the patient's aftercare.
- Every patient in hospital should be reviewed prior to planed discharge, in order to determine what level of aftercare will be necessary to enable him or her to live safely in the community.
- When the discharge plan has been agreed with the patient, carer, and all care team Parties will receive a copy of the discharge care plan.
- The provisional discharge date should be negotiated between the multidisciplinary team and the client (and where appropriate the client's relatives/carers). It is important that the provisional discharge date is identified far enough in advance to permit necessary arrangements to be made and required meetings to take place.
- Documentation of discharge planning will include completed discharge instructions with patient name and signature, documentation of the patients cognitive intactness, and documentation that the patient understand and agrees with the discharge plan, including medications and follow-up care.

## 8. Stages of Discharge Process

### 8.1. Initial Discharge Planning Meeting

- Discharge planning should begin as soon as possible, but preferably no later than the second ward round (multidisciplinary meeting).
- The initial and subsequent meetings at which discharge is being planned should be attended by psychiatric, client, and carer.
- The length of time between the initial discharge planning meeting and the final discharge planning meeting will be dependent upon the progress of the patient.
- Multidisciplinary team should maintain regular contact with each other concerning the patient's progress. An appropriate date for a final discharge meeting should be jointly agreed as soon as possible.

### 8.2. Discharge Planning Meeting(s)

- The treating physician is responsible for determining clinical stability for discharge and identifying posthospitalization medical needs.
- A social worker will conduct a social services needs assessment for homeless inpatients.
- Discharge planning meetings must include an appropriate nursing care plane and discharge plane (see Tables 1, 2 and 3; an applicable care plane and discharge plane format).
- The discharge planning checklist (see Tables 1, 2 and 3; an applicable discharge planning checklist) should be used at this meeting as guidance.

### 8.3. Leaving from Hospital and Day of Discharge

- Leave from hospital should be planned through the ward round (multidisciplinary meetings) in consultation with community staff after discussion with patient and carers, where appropriate.
- If, on the day of discharge, the patient is considered by nursing staff to be fit to leave hospital.
- For patients who are leaving the hospital the discharge checklist should be used to ensure that all identified requirements relating to the day of discharge should be completed. A copy of discharge checklist and care plan should be given to the patient or carer. The discharge checklist will provide a framework for considering the practicalities of a patient going on leave.
- Psychiatric nurse communicates to the client that he is being discharged and ensures they have a copy of the care plan. Assigned nurse also ensure that the client understands their after care arrangements and this process.
  - The assigned nurse gives the client the discharge medication, ensures that the client understands the medication regime, and knows how to obtain the next prescription.
- The assigned nurse returns any stored property to the clients.

## 9. Evaluation of Application Days

In this semester, the main goal was to develop evidence-based guidelines could be applied in the national center of psychiatric health. The topic of discharge planning was chosen to be under study based on significance of discharge planning in psychiatric field. Discharge planning is the best solution to rapid psychiatric clients discharge, decrease institution based, and decrease governments cost.

This literature review was conducted systematically and comprehensively and based on research site of Pub Med, Medline, CINAHL, and Science Direct. The search was also conducted for clinical practice guidelines based on different levels of evidence such as systematic reviews and meta-analyses; randomized controlled trials; controlled trials, cohort or case-control analytic studies; case series: post test only, pretest/posttest; expert opinion including literature/narrative reviews, consensus statements, descriptive studies, and individual case studies.

To evaluate the application of these guidelines, the scale of psychiatric recovery was used. Application of discharge planning in the national center of psychiatric health was encountered with barriers. These barriers as insufficient clinical days per week that being on shift one or two times per week not enough to follow clients' progress, also most client discharge on Sunday and this day we are in university for lectures, so the third stage of discharge planning (leaving from hospital and discharge day) was not applied mostly, also some clients were discharged during the second stage of regular meeting.

Generally, full discharge planning was applied on three clients and psychiatric recovery scale was measured for them before and after. The result demonstrated an improvement in discharge process but three clients are insufficient to generalize the result. So, discharge planning should be applied on other clients. 

## Figures and Tables

**Table 1:** 

Nursing care plan				
Description (aim)		Date set	Review date	Outcome
				
				
				
				
				

Goals and measures	Goal type (Long-term/short-term)	Date set	Review date	Outcome

(1)				□ Achieved
				□ Partially-achieved
				□ Not

(2)				□ Achieved
				□ Partially-achieved
				□ Not

(3)				□ Achieved
				□ Partially-achieved
				□ Not achieved

(4)				□ Achieved
				□ Partially-achieved
				□ Not

Action plan/strategies				

				
				
				
				
				
				
				

Progress notes				

Note changes in client needs and circumstances and changes to care plan.				
				
				
				
				

**Table 2:** 

Discharge plan			
Include role of client, family, community, other agencies and resources			
			
			
			
			
			
			

Date of closure		Initiated by:	

Reason for closure			
		

Goals achieved			
		

Completion of goals			
		

Caregiver satisfaction survey	Is survey conducted; level of caregiver satisfaction/comments		
		

Duration of stay (days)			

Organisation referred for followup			

Staff responsible for followup			

Date of planned followup			

Name of staff and contact details given to client		Tel:	
		Email:	

Client's signature/Date			

Case manager's signature/Date			

**Table 3:** 

Discharge plan checklist	Yes	No
(1) The client's strengths, needs, abilities, and preferences (SNAP) at the point prior to discharge are documented.	( )	( )
(2) The gains from goals achieved are documented.	( )	( )
(3) The likely postdischarge needs and issues are identified and conveyed to client and caregiver.	( )	( )
(4) Referral to other agencies for post-discharge needs is made, where necessary.	( )	( )
(5) Caregivers are briefed on client needs, and informed with other resources available, including caregiver support groups, respite services, and other community resources.	( )	( )
(6) Contact details of a staff from the discharging organization have been given to client and caregiver.	( )	( )
(7) Assigned staff and social worker had arranged to follow up with the client and caregiver, within a specified time-frame.	( )	( )
(8) Information resources, such as pamphlets of community-based services, health-related information (disease prevention, nutrition or diet, coping skills for caregivers, etc.) had been given to client and caregiver.	( )	( )
